# Morphometric assessment of the posterior cranial fossa and its contents in patients with chiari malformation type I and type 0

**DOI:** 10.1007/s00701-026-06878-4

**Published:** 2026-04-16

**Authors:** Busra Candan, Birol Ozkal, Esra Top

**Affiliations:** 1 School of Medicine, Department of Anatomy, Alanya Alaaddin Keykubat University, Alanya, Turkey; 2 School of Medicine, Department of Brain and Neurosurgery, Alanya Alaaddin Keykubat University, Alanya, Turkey

**Keywords:** Chiari malformation, Chiari Type 0, Chiari Type I, Posterior cranial fossa, Morphometry, Neurosurgical anatomy

## Abstract

**Background:**

Chiari Malformation Type I and Type 0 are congenital malformations diagnosed by MRI findings of at least 5 mm and less than 3 mm of cerebellar ectopy below the foramen magnum respectively. In this study we aimed that to comparatively analyze the morphometry of the clivus, tentorium, and posterior cranial fossa in patients with Chiari malformation Types I and 0 versus healthy subjects, and to assess the potential contribution of these measurements to diagnostic accuracy.

**Methods:**

In this study mid-sagittal MRI scans of 326 individuals obtained between 2018–2025 and including patients with Chiari Type I malformation (*n* = 111), Chiari Type 0 malformation (*n* = 27), and age- and sex-matched healthy controls (*n* = 188), were retrospectively analyzed. Multiple cranial base, posterior cranial fossa, cerebellar, and intracranial morphometric parameters and area-based ratios were measured on mid-sagittal T1-weighted images.

**Results:**

We observed that, compared with Chiari Type 0 patients and controls, Chiari Type I patients exhibited a significantly shorter clivus, reduced posterior cranial fossa area, and increased cerebellar area, whereas foramen magnum diameter and total brain area were comparable among groups. Ratio-based analyses demonstrated significantly higher cerebellum-to–posterior fossa and cerebellum-to–brain area ratios in Chiari Type I, indicating increased relative cerebellar occupancy despite preserved global cranial proportions.

**Conclusion:**

Chiari Type I malformation is characterized by specific cranial base remodeling. These morphometric alterations are absent in Chiari Type 0, which remains anatomically comparable to healthy controls. These findings suggest that CM-I is associated with distinct morphoanatomical features, whereas CM-0 does not demonstrate a consistent structural substrate and may represent a functionally defined condition.

## Introduction

Chiari malformations are congenital anomalies of the craniovertebral junction related to the position of the cerebellum and brainstem at the skull base, characterized by herniation of the cerebellar tonsils below the foramen magnum and often accompanied by brainstem descent [21]. Among the various subtypes, Chiari malformation Type I (CM-I) is the most described form [29]. CM-I is defined as caudal herniation of the cerebellar tonsils or the medulla oblongata at least 5 mm below the level of the foramen magnum into the cervical spinal canal [7].

In recent years, the use of cerebellar tonsillar herniation as an absolute diagnostic criterion has become increasingly controversial. In particular, the identification of patients who exhibit significant neurological symptoms and syringomyelia despite the absence of substantial cerebellar tonsillar herniation has demonstrated that the pathophysiology of Chiari malformations cannot be explained solely by cerebellar tonsillar position [8, 13, 23]. Within this context, Chiari malformation Type 0 (CM-0) has been described as a variant in which patients exhibit clinical symptoms comparable to those of CM-I and may present with associated syringomyelia, despite cerebellar tonsillar herniation measuring less than 3 mm or being absent [2, 3]. These findings suggest that the underlying pathology is not limited to cerebellar tonsillar displacement alone but also involves a reduction in posterior fossa volume, alterations in craniovertebral junction morphology, and microscopic or functional impairment of cerebrospinal fluid flow at the level of the foramen magnum [3, 22].

However, it should be noted that Chiari malformation type 0 (CM-0) remains a controversial entity and is not universally accepted as a distinct diagnostic category. In this context, the CM-0 group in the present study should be interpreted as a clinically defined cohort characterized by Chiari-like symptoms and minimal tonsillar descent, rather than a universally established nosological entity [2, 3].

Posterior fossa hypoplasia has been shown to cause cerebrospinal fluid flow disturbances and syringomyelia even in the absence of cerebellar tonsillar herniation [20, 26]. Clivus length, a key component of posterior fossa morphometry, serves as an indicator of cranial base development and plays a critical role in the pathogenesis of Chiari malformations. A shortened clivus leads to a reduction in the anteroposterior depth of the posterior fossa, resulting in relative crowding of the brainstem and cerebellar structures [13]**.** In CM-0, posterior fossa insufficiency related to subtle cranial base alterations has been proposed as a potential contributor to symptom development, even in the absence of significant tonsillar descent [18, 20, 22].

Accordingly, the aim of the present study was to perform a comprehensive, landmark-based morphometric assessment of the cranial base, tentorium, and posterior cranial fossa complex in patients with CM-I and CM-0, as well as in age- and sex-matched healthy control subjects using mid-sagittal MRI. By evaluating both absolute measurements and ratio-based parameters, this study seeks to determine whether CM-I and CM-0 exhibit distinct morphoanatomical profiles and to assess the potential value of quantitative morphometry beyond cerebellar tonsillar descent in radiological and surgical evaluation.

## Materials and methods

In this retrospective study, patient data obtained from the Department of Neurosurgery at Alanya Alaaddin Keykubat University between 2018 and 2025 were analyzed. A total of 111 patients diagnosed with CM-I and 27 patients diagnosed with CM-0 who were admitted to the neurosurgery department were included in the study. In addition, 188 age- and sex-matched individuals who underwent MRI for non-specific clinical complaints (such as headache or dizziness) and demonstrated no structural abnormalities on imaging were included as the control group. Although these individuals did not exhibit structural pathology, they may not represent completely healthy volunteers. Individuals aged 18 years or older with complete and high-quality magnetic resonance imaging scans of the brain and cervical spine were included in the study. Patients classified as CM-0 were identified based on a combination of clinical and radiological criteria. CM-0 was defined as the presence of Chiari malformation–like clinical symptoms in the absence of significant cerebellar tonsillar herniation on magnetic resonance imaging. Specifically, patients were included in the CM-0 group if cerebellar tonsillar descent was less than 3 mm below the foramen magnum on mid-sagittal MRI, measured relative to the McRae line. Patients presented with symptoms commonly reported in association with Chiari malformation, such as occipital headache, neck pain, Valsalva-induced symptoms, or other brainstem-related complaints. The presence of syringomyelia was allowed but not mandatory for inclusion, consistent with prior descriptions of CM-0. Patients with cerebellar tonsillar descent of 5 mm or greater were classified as CM-I. Additional exclusion criteria for all groups included posterior fossa mass lesions, hydrocephalus, basilar invagination, craniovertebral junction anomalies, prior craniospinal surgery, or significant imaging artifacts that could compromise morphometric measurements. This classification approach is consistent with previously proposed descriptions of CM-0, which emphasize functional cerebrospinal fluid disturbance rather than overt structural hindbrain herniation. Treatment strategies were not standardized within the scope of this retrospective study, and management decisions were based on individual clinical evaluation.

Mid-sagittal T1-weighted brain magnetic resonance images, which represent a standard imaging modality for the evaluation of Chiari malformations, were analyzed in all groups. All images were acquired using a 1.5-T Siemens Magnetom Altea scanner (Siemens Healthineers, Erlangen, Germany). Image evaluation was independently performed by two anatomists using Sectra IDS7 software (version 24.2.16.6066, 2023). Observers were blinded to clinical group allocation. Imaging parameters included a slice thickness of 3 mm, an interslice gap of 0.5 mm, a field of view of 230–280 mm, and a matrix size of 205 × 256 for T1-weighted images and 320 × 320 for T2-weighted images. For T1-weighted sequences, the repetition time was approximately 250 ms with an echo time of 4.76 ms, whereas for T2-weighted sequences, the repetition time ranged from 3000 to 4000 ms with an echo time of 100–120 ms.

**Fig. 1:**
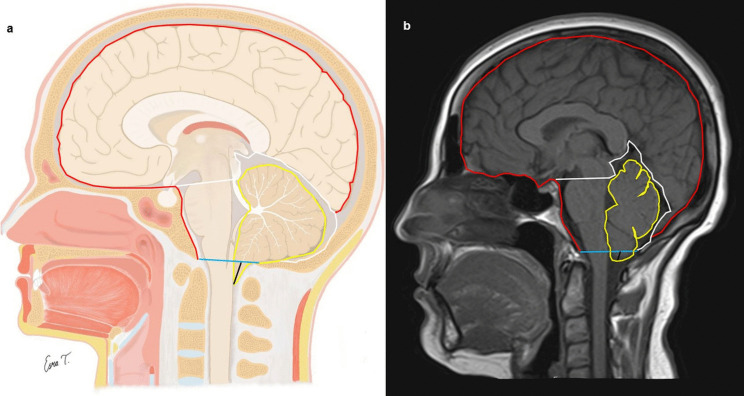
**a** Schematic illustration in the mid-sagittal plane demonstrating the defined morphometric boundaries used for analysis. **b** Corresponding mid-sagittal T1-weighted magnetic resonance image. Colored lines indicate the following measurements: intracranial cavity area and perimeter (red), cerebellar area and perimeter (yellow), posterior cranial fossa area and perimeter (white), anteroposterior diameter of the foramen magnum (blue), and degree of cerebellar tonsillar descent (black). Consistent color coding is maintained across both panels to ensure anatomical correlation

**Fig. 2:**
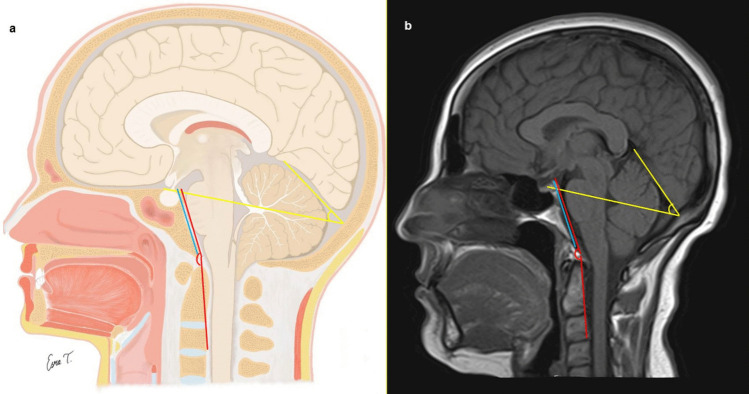
**a** Schematic illustration in the mid-sagittal plane demonstrating angular and linear morphometric measurements. **b** Corresponding mid-sagittal T1-weighted magnetic resonance image. Colored markings indicate the clival angle (red), tentorial angle (yellow), and clivus length (blue). Consistent color coding is maintained across both panels to ensure anatomical correlation

The following parameters were measured for all individuals in the midsagittal slice: the clivus length, the clivus angle, the tentorial angle, anteroposterior diameter of the foramen magnum, degree of tonsillar descent (for CM-0 and CM-I), area and perimeter of the posterior fossa, area and perimeter of the cerebellum, area and perimeter of the intracranial cavity (Figs. 1 and 2). Ratios of cerebellar area to posterior fossa area, posterior fossa area to intracranial area, and cerebellar area to intracranial area were also calculated.

The clivus length (mm) was measured as the linear distance between the basion and the dorsum sellae (Fig. 2). The clivus angle (Wackenheim’s clivus angle) was measured as the angle between the line drawn from the dorsum sellae to the basion and the line extending from the basion to the posterior surface of the C2 vertebral body (Fig. 2). The tentorial angle was measured as the angle between the tentorium and a line drawn from the internal occipital protuberance to the tuberculum sellae (Twining’s line) (Fig. 2). The anteroposterior diameter of the foramen magnum was determined by measuring the distance between the basion, located on the anterior margin, and the opisthion, located on the posterior margin of the foramen magnum (Fig. 1).

### Statistical analysis

All statistical analyses were performed using IBM SPSS Statistics for Windows, Version 26.0 (IBM Corp., Armonk, NY, USA). Continuous variables were expressed as mean ± standard deviation (SD), whereas categorical variables were presented as frequencies and percentages. The normality of data distribution was assessed using the Shapiro–Wilk test. As several morphometric variables deviated from a normal distribution, nonparametric statistical methods were applied throughout the analysis. Comparisons among the three study groups (CM-I, CM-0, and healthy controls) were conducted using the Kruskal–Wallis test. When a statistically significant difference was detected, pairwise post hoc comparisons were performed using the Mann–Whitney U test with Bonferroni correction to adjust for multiple comparisons. In addition to absolute morphometric measurements, ratio-based parameters were calculated to evaluate relative spatial relationships between the cerebellum, posterior cranial fossa, and total brain area. These ratios included the cerebellum to posterior cranial fossa area ratio, cerebellum to intracranial area ratio, and posterior cranial fossa to intracranial area ratio. Ratio-based comparisons among groups were analyzed using the same nonparametric statistical approach applied to absolute measurements. Interobserver reliability for all morphometric measurements was assessed using the intraclass correlation coefficient (ICC) based on a two way random effects model with absolute agreement. ICC values were interpreted according to established criteria, with values > 0.75 indicating good reliability and values > 0.90 indicating excellent reliability. Interobserver reliability analysis demonstrated good to excellent agreement across all morphometric parameters, with ICC values ranging from 0.82 to 0.94, supporting the robustness and reproducibility of the morphometric measurements. All statistical tests were two-tailed, and a *p* value < 0.05 was considered statistically significant. A post hoc power analysis was performed for the primary morphometric outcomes using the observed effect sizes and sample sizes of the study groups. The analysis indicated that the study had sufficient statistical power (power > 0.80) to detect significant intergroup differences.

## Result

A total of 326 subjects were included in the study, comprising patients with CM-I (*n* = 111), CM-0 (*n* = 27), and healthy controls (*n* = 188). There were no statistically significant differences among the groups with respect to age or sex distribution (*p* > 0.05). Demographic characteristics of the study groups are summarized in Table 1.

**Table 1:** Demographic characteristics of the study groups

Group	*n*	Female, *n* (%)	Male, *n* (%)	Age (mean ± SD)
CM-I	111	74 (66.7%)	37 (33.3%)	40.3 ± 12.8
CM-0	27	12 (44.4%)	15 (55.6%)	45.6 ± 15.7
Control	188	110 (58.5%)	78 (41.5%)	43.5 ± 17.2

Comparative morphometric analyses were performed across the three study groups (Table 2). The tentorial angle demonstrated a decreasing trend from CM-I to control subjects, whereas the clival angle did not differ significantly among groups. Clivus length was shortest in the CM-I group, with progressively greater values observed in the CM-0 and control groups. The posterior cranial fossa area was significantly smaller in CM-I patients, while the cerebellar area was significantly larger in this group compared with CM-0 patients and healthy controls. In contrast, foramen magnum diameter and total intracranial area showed no significant intergroup differences. Post hoc pairwise comparisons using Bonferroni correction demonstrated that the significant differences were consistently driven by comparisons involving the CM-I group (Table 3). Specifically, CM-I patients differed significantly from both CM-0 patients and healthy controls in terms of clivus length, posterior cranial fossa area, and cerebellar area (all *p* < 0.05 after correction), whereas no significant differences were observed between CM-0 patients and controls for these parameters.

**Table 2:** Comparison of measured morphometric parameters between groups

Parameter	CM-I	CM-0	Control	*p*-value
Tentorial angle (°)	37.9 ± 7.3	36.5 ± 5.9	35.0 ± 5.4	0.002
Clival angle (°)	166.1 ± 9.4	168.8 ± 8.2	166.6 ± 8.5	0.450
Clivus length (mm)	39.4 ± 6.0	42.4 ± 4.6	43.5 ± 4.5	< 0.001
Foramen magnum diameter (mm)	35.2 ± 4.2	35.7 ± 3.9	35.0 ± 3.1	0.462
Posterior fossa area (mm^2^)	2961 ± 316	3125 ± 319	3172 ± 897	< 0.001
Cerebellar area (mm^2^)	1429 ± 172	1217 ± 189	1245 ± 199	< 0.001
Brain area (mm^2^)	14,691 ± 1350	14,532 ± 984	14,587 ± 1060	0.642

*P*-values represent overall comparisons using the Kruskal–Wallis test. Significant results were further explored with Bonferroni-corrected post hoc pairwise comparisons (see Table 3)

**Table 3:** Bonferroni-corrected post hoc comparisons of parameters with significant overall group differences

Parameter	Type	CM-I vs. CM-0	CM-I vs. Control	CM-0 vs. Control
Tentorial angle	Absolute	0.392	< 0.001	0.222
Clivus length	Absolute	0.011	< 0.001	0.319
Posterior cranial fossa area	Absolute	0.018	< 0.001	0.893
Cerebellar area	Absolute	< 0.001	< 0.001	0.552
Cerebellum/Posterior fossa	Ratio	< 0.001	< 0.001	0.42
Cerebellum/Brain	Ratio	< 0.001	< 0.001	0.31

Only parameters with significant overall group differences (Kruskal–Wallis test) are included. *P*-values are adjusted using Bonferroni correction

**Fig. 3:**
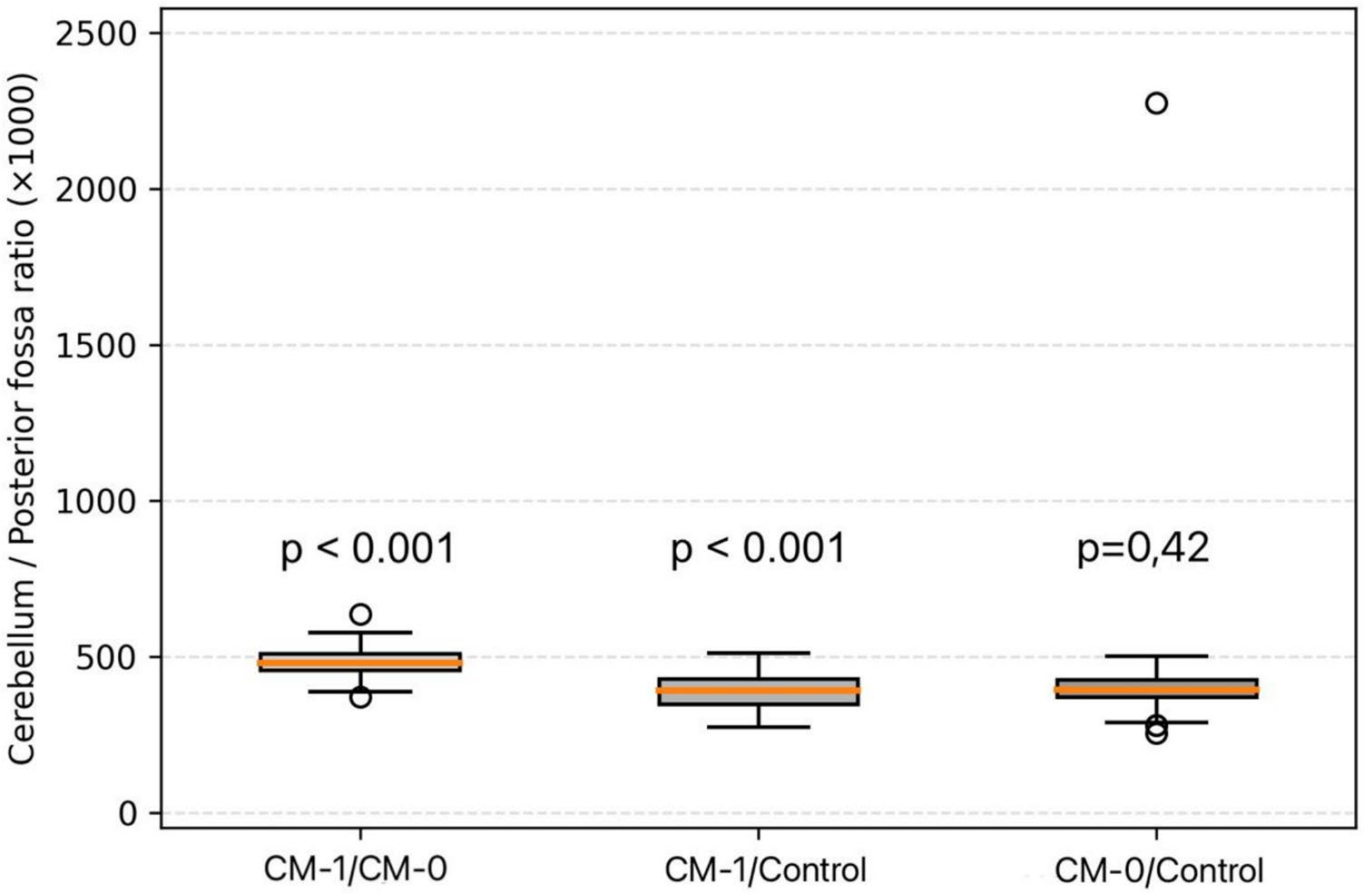
Boxplot illustrating the cerebellum-to–posterior fossa area ratio (× 1000) across study groups. The ratio was significantly higher in Chiari type I compared with both Chiari 0 and healthy controls (Bonferroni-corrected *p* < 0.001 for both), whereas no significant difference was observed between Chiari 0 and controls (ns)

**Fig. 4:**
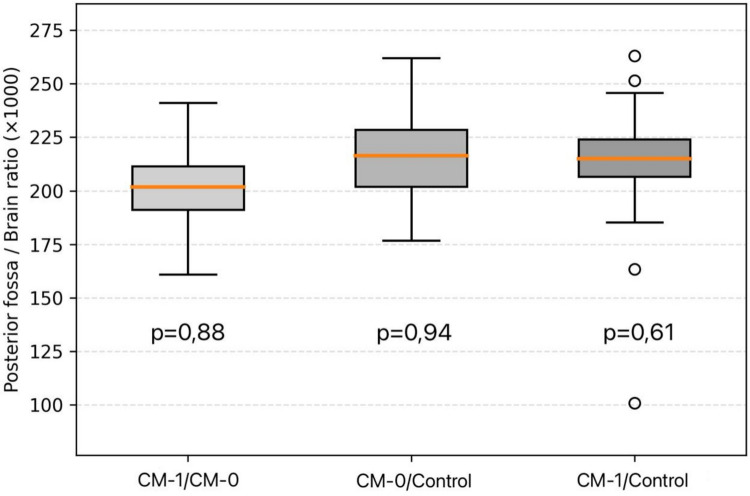
Boxplot illustrating the posterior fossa to brain area ratio (× 1000) across study groups. No significant differences were detected among groups, indicating preservation of global posterior fossa proportion relative to total brain area

Ratio-based analyses were conducted to evaluate relative spatial relationships between the cerebellum, posterior cranial fossa, and intracranial cavity (Table 4). The posterior cranial fossa to intracranial area ratio did not differ significantly among groups. In contrast, both the cerebellum to posterior cranial fossa area ratio and the cerebellum to intracranial area ratio demonstrated significant intergroup differences, with higher values observed in CM-I patients compared with CM-0 patients and healthy controls (Figs. 3 and 4).

**Table 4:** Ratio-Based Morphometric Measurements Across Study Groups

Ratio parameter	CM-I	CM-0	Control	*p*-value
Cerebellum/Posterior fossa	483.8 ± 44.4	391.2 ± 60.4	430.3 ± 39.3	< 0.001
Cerebellum/Brain	97.5 ± 10.7	83.8 ± 12.6	85.7 ± 14.4	< 0.001
Posterior fossa/Brain	202.0 ± 16.2	215.3 ± 19.0	217.8 ± 21.4	0.57

Sex-stratified analyses were conducted to evaluate whether the morphometric patterns distinguishing CM-I from CM-0 and healthy controls were preserved within female and male subgroups.

Among female subjects, statistically significant intergroup differences were observed for tentorial angle, clivus length, posterior cranial fossa area, cerebellar area, and the cerebellum-to–posterior cranial fossa area ratio. Female patients with CM-I demonstrated shorter clivus length, reduced posterior cranial fossa area, and higher relative cerebellar occupancy compared with CM-0 patients and healthy controls (Table 5).

**Table 5:** Comparison of measured morphometric parameters between groups in female patients

Parameter	CM-I (n = 74)	CM-0 (n = 12)	Control (n = 110)	*p*-value
Tentorial angle (°)	39.15 ± 7.26	36.47 ± 5.61	35.77 ± 5.77	0.014
Clivus length (mm)	37.22 ± 5.18	39.35 ± 3.98	41.87 ± 3.81	< 0.001
Posterior fossa area (mm^2^)	2877 ± 260	2960 ± 290	3025 ± 270	< 0.001
Cerebellar area (mm^2^)	1412 ± 170	1202 ± 186	1231 ± 191	0.042
Cerebellum/PF ratio	490.1 ± 44.8	406.1 ± 60.5	430.3 ± 39.3	< 0.001

Among male subjects, a similar directional pattern was observed. Male patients with CM-I exhibited shorter clivus length, increased cerebellar area, and higher cerebellum-to–posterior cranial fossa area ratios compared with CM-0 patients and controls. Although some parameters did not reach statistical significance, likely due to smaller subgroup sizes, particularly within the CM-0 cohort, the overall morphometric profile was consistent with that observed in the primary group-based analyses (Table 6).

**Table 6:** Comparison of measured morphometric parameters between groups in male patients

Parameter	CM-I (*n* = 37)	CM-0 (*n* = 15)	Control (*n* = 78)	*p*-value
Tentorial angle (°)	38.32 ± 7.38	35.60 ± 6.48	34.92 ± 5.22	0.091
Clivus length (mm)	41.26 ± 6.11	44.30 ± 5.44	45.07 ± 4.89	0.001
Posterior fossa area (mm^2^)	3131 ± 354	3257 ± 284	3271 ± 261	0.140
Cerebellar area (mm^2^)	1463 ± 158	1229 ± 164	1267 ± 141	< 0.001
Cerebellum/PF ratio	469.6 ± 42.9	378.5 ± 48.5	388.9 ± 43.5	< 0.001

## Discussion

The present study provides a comprehensive morphometric assessment of the clivus–tentorium–posterior cranial fossa complex, which refers to the integrated anatomical relationship between cranial base structures and posterior fossa geometry, across CM-I, CM-0, and healthy control groups. The primary finding is that morphometric alterations were predominantly observed in patients with CM-I, whereas individuals with CM-0 exhibited cranial base measurements largely comparable to those of healthy controls. Specifically, CM-I patients demonstrated a significantly shorter clivus, reduced posterior cranial fossa area, altered tentorial orientation, and increased cerebellar area compared with both CM-0 patients and controls. These findings are consistent with previous studies [1, 5, 12, 18]. In contrast, CM-0 patients showed no significant differences from controls in these absolute morphometric parameters. Importantly, total brain area did not differ among groups, indicating that the observed changes reflect localized cranial base remodeling rather than global cerebral enlargement. These findings support the interpretation that CM-I is characterized by region-specific structural alterations of the posterior cranial fossa, rather than a generalized abnormality of brain size or growth. Prior morphometric and volumetric studies have similarly reported clival hypoplasia and posterior fossa insufficiency as defining features of CM-I [5, 12, 17, 20, 26]. However, it is important to emphasize that Chiari malformation type I is not a uniform entity, and the morphometric features identified in this study should not be interpreted as pathognomonic. Increasing evidence suggests that CM-I represents a heterogeneous condition, sometimes referred to as a “Chiari syndrome,” encompassing a spectrum of underlying pathophysiological mechanisms rather than a single anatomical abnormality. In this context, cerebellar tonsillar ectopy may represent a secondary consequence rather than the primary driver of disease in certain patients. Importantly, the existing literature does not present a uniform consensus regarding the morphometric characteristics of Chiari malformations. Several studies have emphasized that CM-I represents a heterogeneous condition rather than a single anatomical entity and may be better conceptualized as a “Chiari syndrome” encompassing multiple pathophysiological mechanisms [6, 24]. In this context, it has been reported that not all patients with CM-I exhibit consistent posterior fossa hypoplasia or clival shortening, and that morphometric alterations may vary substantially between individuals [4, 17, 20]. Furthermore, several authors have argued that cerebellar tonsillar ectopia alone is insufficient to explain symptomatology, as significant clinical findings may occur even in the absence of marked tonsillar descent, while some individuals with pronounced herniation remain asymptomatic [16, 23]. In addition, the role of cerebrospinal fluid (CSF) flow dynamics has been increasingly emphasized, particularly in CM-0 patients, where functional disturbances rather than structural abnormalities may play a dominant role in symptom development [2, 11, 22]. Taken together, these discrepancies in the literature suggest that morphometric findings should be interpreted with caution and considered as supportive rather than definitive diagnostic markers, in line with the findings of the present study. The present results should be viewed within the broader context of an ongoing debate, where both structural and functional factors contribute to the pathophysiology of Chiari-related disorders. The morphometric alterations identified in the present study should be considered supportive rather than diagnostic, and should be interpreted in conjunction with clinical findings and other imaging features. This perspective is consistent with previous studies highlighting the variability and multifactorial nature of Chiari-related disorders [6, 24, 28]. Post hoc analyses further clarified that the significant intergroup differences consistently arose from comparisons involving the CM-I group, while no meaningful morphometric separation was observed between CM-0 and control subjects. Importantly, the absence of significant morphometric differences between CM-0 patients and healthy controls raises fundamental questions regarding the validity of CM-0 as a distinct morphoanatomical entity. This finding suggests that CM-0 may represent a functional condition rather than a structurally defined disorder, with diagnosis relying primarily on clinical presentation and cerebrospinal fluid dynamics rather than measurable anatomical alterations. In contrast, these results reinforce the interpretation of CM-I as a distinct morphoanatomical entity characterized by clival shortening and posterior fossa insufficiency. The lack of structural differences between CM-0 and controls further supports the notion that CM-0 does not share the cranial base remodeling traditionally attributed to Chiari type I and may instead reflect functional cerebrospinal fluid disturbances in the absence of overt structural abnormalities [11, 16, 22]. The lack of consistent cerebrospinal fluid flow assessment may be particularly relevant in CM-0 patients, where functional disturbances rather than structural abnormalities have been proposed. In this context, the CM-0 group in the present study should be interpreted with caution, as it represents a clinically defined subset rather than a distinct morphoanatomical diagnosis. Accordingly, the findings of the present study support a more cautious interpretation of CM-0 as a clinical rather than morphoanatomical entity.

Previous morphometric studies have reported inconsistent findings regarding tentorial orientation in CM-I [12, 14, 17, 20]. In contrast, other investigations have identified a reduced or flatter tentorial angle in CM-I patients, suggesting that different patterns of tentorial orientation may influence posterior fossa geometry via distinct biomechanical mechanisms [1, 22]. Collectively, these divergent findings indicate that tentorial angle alteration in CM-I is not uniform but rather represents a variable component of the broader cranial base remodeling process, reinforcing the need for individualized, quantitative morphometric assessment rather than reliance on a single geometric parameter. The present study adds to this literature by demonstrating that tentorial angle differences were primarily evident in comparisons between CM-I patients and healthy controls, whereas CM-0 patients exhibited tentorial measurements comparable to controls. This pattern suggests that tentorial reorientation may represent a secondary or adaptive morphological response associated with established CM-I cranial base remodeling, rather than a universal feature across the Chiari spectrum.

Beyond absolute measurements, ratio-based analyses provided important mechanistic insights into the spatial relationships between neural tissue and the posterior cranial compartment. While the posterior fossa to brain area ratio remained preserved across all groups, both the cerebellum to brain and cerebellum to posterior fossa area ratios were significantly increased in CM-I patients compared with CM-0 and healthy controls. The preservation of the posterior fossa to brain ratio indicates that global cranial proportions and overall brain size remain balanced. In contrast, the altered cerebellum-related ratios reflect relative cerebellar crowding within a morphologically restricted posterior fossa [9]. These findings support existing models proposing that CM-I arises from a spatial mismatch between normally developed neural tissue and an underdeveloped posterior cranial fossa, rather than from excessive cerebellar growth [17, 20, 25]. In contrast, the lack of significant ratio differences between CM-0 patients and controls further supports the absence of overt morphometric crowding in CM-0, reinforcing its distinction from CM-I.

Sex-stratified analyses demonstrated that the morphometric features characterizing CM-I malformation were preserved across both female and male subgroups. The persistence of shortened clivus length, posterior cranial fossa reduction, and increased relative cerebellar occupancy in both sexes indicates that these alterations are disease-specific rather than sex-dependent [10, 15]. Although some morphometric parameters showed attenuated statistical significance in male patients, this finding is most plausibly attributable to reduced subgroup sizes rather than true sex-dependent anatomical differences. Importantly, CM-0 patients of both sexes consistently exhibited morphometric measurements comparable to those of healthy controls, further reinforcing the distinction between the structural remodeling observed in CM-I and the absence of such features in CM-0 [2]. Collectively, these sex-stratified results strengthen the interpretation that posterior cranial fossa remodeling represents a sex-independent anatomical hallmark of CM-I and support the utility of quantitative morphometric assessment beyond cerebellar tonsillar descent alone. The consistency of these findings across sexes enhances the robustness and generalizability of the study’s primary conclusions.

The present findings have important implications for the diagnosis and management of Chiari related disorders. The confinement of structural cranial base remodeling to CM-I patients underscores the necessity of detailed morphometric assessment during preoperative evaluation [22, 27]. Reliance solely on cerebellar tonsillar descent or clinical symptoms may obscure critical anatomical distinctions and lead to inappropriate surgical decision-making. The demonstrated association between shortened clivus length, reduced posterior cranial fossa area, and cerebellar crowding in CM-I supports the anatomical rationale for posterior fossa decompression in appropriately selected patients [4, 19, 27, 29]. However, these findings should be interpreted with caution, as they are not universally present in all CM-I patients. Conversely, the absence of comparable morphometric alterations in CM-0 suggests that routine decompression may not be universally justified highlighting the importance of individualized treatment strategies informed by quantitative morphometric assessment. Furthermore, the preservation of total brain area across all groups emphasizes the importance of focusing on regional cranial morphology rather than global brain size during radiological evaluation. Incorporating standardized measurements of clivus length, tentorial orientation, and posterior cranial fossa dimensions into routine imaging protocols may enhance diagnostic accuracy and risk stratification in patients with suspected Chiari malformation.

## Limitations

Several limitations of the present study should be acknowledged. First, the use of two-dimensional mid-sagittal measurements instead of three-dimensional volumetric analysis represents a methodological limitation, as it may not fully capture the complex spatial relationships within the posterior cranial fossa. Future studies incorporating volumetric imaging techniques may provide a more comprehensive evaluation of posterior fossa–cerebellar relationships. Second, the retrospective design inherently limits control over patient selection and imaging protocols. An a priori power analysis was not performed due to the retrospective nature of the study; however, a post hoc power analysis was conducted to assess the adequacy of the sample size. Third, cerebrospinal fluid flow dynamics were not assessed, as phase-contrast MRI was not uniformly available across all patients. Furthermore, phase-contrast MRI data were not available for all patients, including those with CM-0, limiting the evaluation of cerebrospinal fluid flow dynamics. Fourth, certain clinical variables were not consistently recorded across groups. Body mass index (BMI) data were not consistently available and therefore could not be included in the analysis. Finally, the cross-sectional nature of the study precludes assessment of temporal changes in cranial base and posterior cranial fossa morphology., as it may not fully capture the complex spatial relationships within the posterior cranial fossa.

## Conclusion

Chiari type I malformation is associated with distinct cranial base remodeling, characterized by clival shortening, reduced posterior cranial fossa area, and increased relative cerebellar occupancy. In contrast, patients with Chiari type 0 exhibit morphometric features largely comparable to those of control subjects, suggesting the absence of a consistent structural substrate. CM-I demonstrates distinct morphoanatomical features, whereas CM-0 does not exhibit consistent structural differences compared to controls and may represent a functional rather than a structurally defined condition.

## Data Availability

The datasets generated and/or analyzed during the current study are available from the corresponding author on reasonable request.
